# Supplementary material for: Panoramic-reconstruction temporal imaging for seamless measurements of slowly-evolved femtosecond pulse dynamics
Source: Nat Commun. 2017 Jul 5;8:61. doi: 10.1038/s41467-017-00093-7 (PMC5498544; doi:10.1038/s41467-017-00093-7)
Supplement: Supplementary file 1 — Supplementary Information [file 41467_2017_93_MOESM1_ESM.pdf]

File name: Supplementary Information
Description: Supplementary Notes, Supplementary Figures and Supplementary References

Supplementary Note 1: Dispersion and spectral intensity response of the buffer

In this section, the residual dispersion and the spectral intensity response of the optical buffer is characterized, which are responsible for the finite pulse-shape distortion during the buffering. First of all, the residual dispersion is estimated using an intensity auto-correlator and an optical spectrum analyser (OSA). The loop of the optical buffer is opened and a broad-band femtosecond source singly passes the buffer. The corresponding spectral bandwidth and the pulse width are measured to estimate the net dispersion. To measure the dispersion more accurately, the bandpass filter (BPF) is removed first so that a much broader pulse spectrum can pass the buffer, and therefore the pulse width broadening is much more sensitive to the dispersion. As shown in Supplementary Figure 1a, the original pulse had a 3-dB spectral bandwidth of 63 nm. The corresponding transform-limited pulse width is estimated to be about 56 fs based on a Gaussian-shape approximation. After singly passing the optical buffer, the spectral bandwidth is narrowed down to about 37 nm owing to the limited gain bandwidth of erbium-doped fibre (EDF). Similarly, the transform-limited pulse width is approximately 95 fs. The actual pulse width corresponding to the two spectra in Supplementary Figure 1a, *i.e.* before and after passing the optical buffer is measured to be 131 fs and 148 fs, respectively. Based on the above values, the dispersion is estimated using the following equation [1].

$$\Delta t_{\text{out}} = \frac{\sqrt{\Delta t^4 + 16(\ln 2)^2 \Phi_2''^2}}{\Delta t} \tag{1}$$

where Δt refers to the original pulse width, while Δt_{out} is the pulse width after passing through a group-delay dispersion (GDD) Φ_2''. Since the input pulse is not transform-limited, the initial chirp

on the pulse should also be taken into consideration. The largest possible dispersion inside the buffer corresponds to the situation where the initial chirp and the chirp after the buffer have opposite sign. In this case, the GDD of the buffer is calculated to be

$$\Phi_2''\Big|_{\Delta t=56\,\text{fs},\Delta t_{\text{out}}=131\,\text{fs}} + \Phi_2''\Big|_{\Delta t=95\,\text{fs},\Delta t_{\text{out}}=148\,\text{fs}} = 6.12\times10^{-3}\,\text{ps}^2 \qquad (2)$$

which corresponds to the dispersion of about 0.28-m single-mode fibre (SMF). The BPF that is previously taken out of the cavity had a foot print of about 3 cm and the corresponding dispersion is negligible. Under this condition (largest possible dispersion), a transform limited 740-fs pulse, which equals the resolution of the time magnifier, will only be broadened by less than 5% after circulating inside the buffer for ten roundtrips. Therefore, a conclusion can be safely drawn that the influence of net residual dispersion on the fidelity of buffering is negligible.

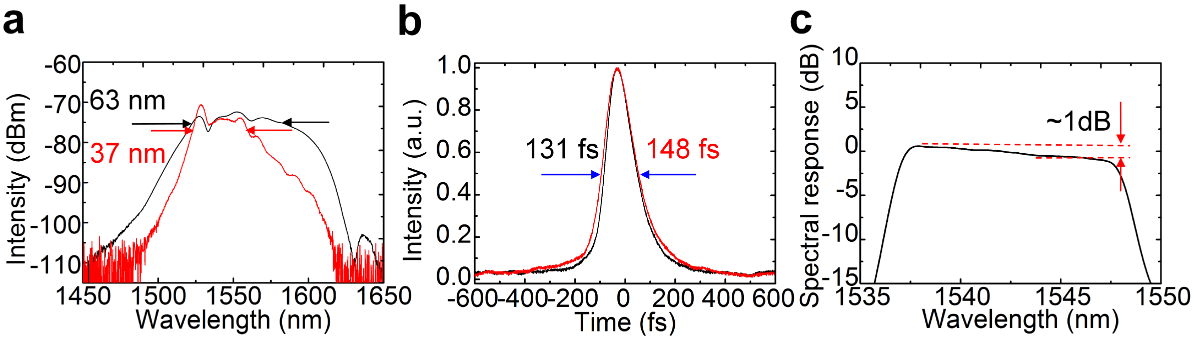

Supplementary Figure 1 | Characterization of the buffer dispersion and spectral intensity response. a, Optical spectra of test pulse before (black) and after (red) single passing the buffer. **b,** Pulse width before (black) and after (red) the buffer. **c,** Spectral response of the buffer.

In addition to the net residual dispersion, the non-ideal spectral intensity response (or gain spectrum) of the buffer may also induce pulse shape distortion. In Supplementary Figure 1c, the

normalized overall spectral intensity response is shown, which is obtained by multiplying the gain spectrum of EDF and the spectral response of the BPF. As shown in the figure, the actual response is slightly tilted and the value at short wavelength side is about 1 dB higher than that at the long wavelength side. The non-uniformity of the EDF gain and the BPF contribute almost equally to the tilted response. As a consequence, the spectral shape of the original waveform will be slightly narrowed after multiple times of buffering. However, as has been shown in the main text Figure 3, for an un-chirped waveform, this tilted spectral intensity response together with distortions from dispersion and amplification noise only cause less than 1% deviation from original envelope after ten circulations. Therefore the current performance of the optical buffer is still acceptable for proof-of-concept demonstration. Ultimately, the optical gain equalizer can be incorporated to the cavity to further improve the performance, if necessary.

Supplementary Note 2: Generation of arbitrary waveform

The arbitrary waveforms in the main text are generated from a continuous-wave (CW) pumped ultrahigh-*Q* microresonator. As shown in Supplementary Figure 2a, the optical spectrum consists of multiple equidistant spectral lines with a spacing about 89 GHz, which has been band-pass filtered to fit the measurement range of the time magnifier. Since the spectral lines are not phase-locked to each other, the spectral phase changes continuously in spite of the stable spectral shape. Therefore, the corresponding waveform is also evolving all the time, which provides the non-repetitive arbitrary waveform as signal-under-test (SUT). The twenty groups of buffered SUTs used to characterize buffering performance in main text Figure 3f are shown in Supplementary Figure 2b. The buffer loads a section of arbitrary waveform from the ultrahigh-*Q* microresonator around every 500 ns and generates ten high-fidelity replicas for each loaded SUT. In

Supplementary Figure 2b, the output waveform from the buffer is recorded for about 10 μs and therefore includes 20 consecutive buffered groups, where a red dashed circle indicates one group. As observed, the buffered waveforms had distinctively different intensities, while in each group, the ten replicas show similar height with finite fluctuation induced by noise. As an example, the zoom-in of the 12th group is shown in Supplementary Figure 2c (same figure as main text Figure 3b).

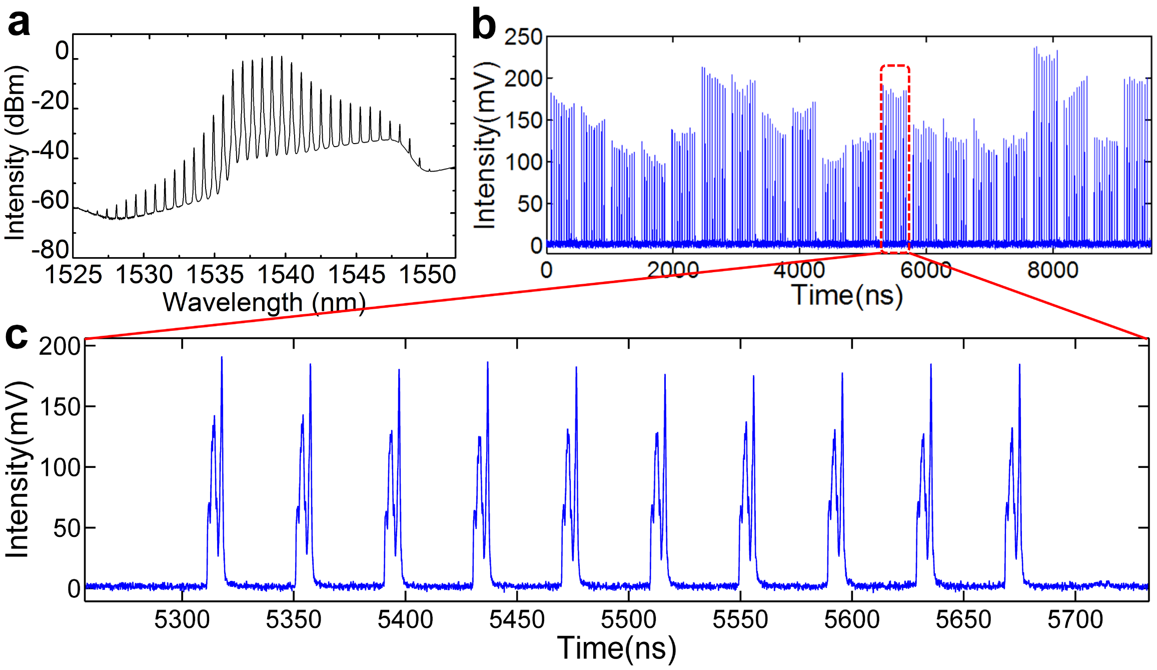

Supplementary Figure 2 | a, Optical spectrum of the constantly-evolving arbitrary waveform. **b,** Waveform showing 20 groups of buffered SUT and each group is separated by 500 ns. **c,** Zoom-in of the 12th group in **b**.

Supplementary Note 3: Waveform stitching process

Intensity calibration

In order to reconstruct a long dynamic waveform from ten mosaic magnified sections, data post-processing is required to calibrate the intensity of each frame of magnified waveform before waveform-stitching can be conducted. Each time a section of SUT is loaded into the system, the PARTI system will output ten sections of magnified waveforms, which corresponds to ten consecutive positions on the SUT, as shown in Supplementary Figure 3b. However, as can be observed on each of the ten frames, the magnified waveform sections show a similar envelope shape even though different SUT positions are measured. This can be understood by looking at the four-wave mixing (FWM) spectrum of the time lens measured after the highly-nonlinear fibre (HNLF), as shown in Supplementary Figure 3a. Spectral components from 1538 nm to 1547 nm are filtered out and launched into the parametric time lens to mix with the swept pump centred at 1560. The idler centres around 1580 is generated, which is filtered out and then goes through output dispersion to generate waveforms in Supplementary Figure 3b. Owing to the non-flat spectrum of the swept pump, as well as the different phase-matching condition of the FWM at different pumping wavelength, the parametric conversion efficiency of the time lens at different input time is actually different, which gives rise to the tilted envelope in each output frame. Intuitively, this is equivalent to a spatial thin lens that has different transmission coefficients at different positions. This will degrade the waveform stitching quality, since the magnified waveforms that correspond to the same position on the SUT may show different intensities in two consecutive output frames, which results in discontinuity at the stitching area. Consequently, intensity calibration according to the time lens responsivity curve is required.

To obtain the responsivity curve of the time lens, we add up 2,500 output frames when the dynamic changing waveform is continuously measured by the PARTI system and the result is shown in Supplementary Figure 3c. Since 250 totally different sections of SUT have been

measured, the envelope shown in Supplementary Figure 3c should be safely attributed to the responsivity of the time lens. After performing further digital smoothing and normalization, the responsivity curve is obtained, as shown in Supplementary Figure 3d. With the calibration curve, we are able to perform intensity calibration to individual output frames from the system. Supplementary Figure 3e shows the zoom-in view of one frame in Supplementary Figure 3b, and the overall envelope matches well with the responsivity curve we obtain. After the calibration, the reshaped waveform is shown in Supplementary Figure 3f, which shows much better intensity uniformity and can thus be further used for waveform stitching.

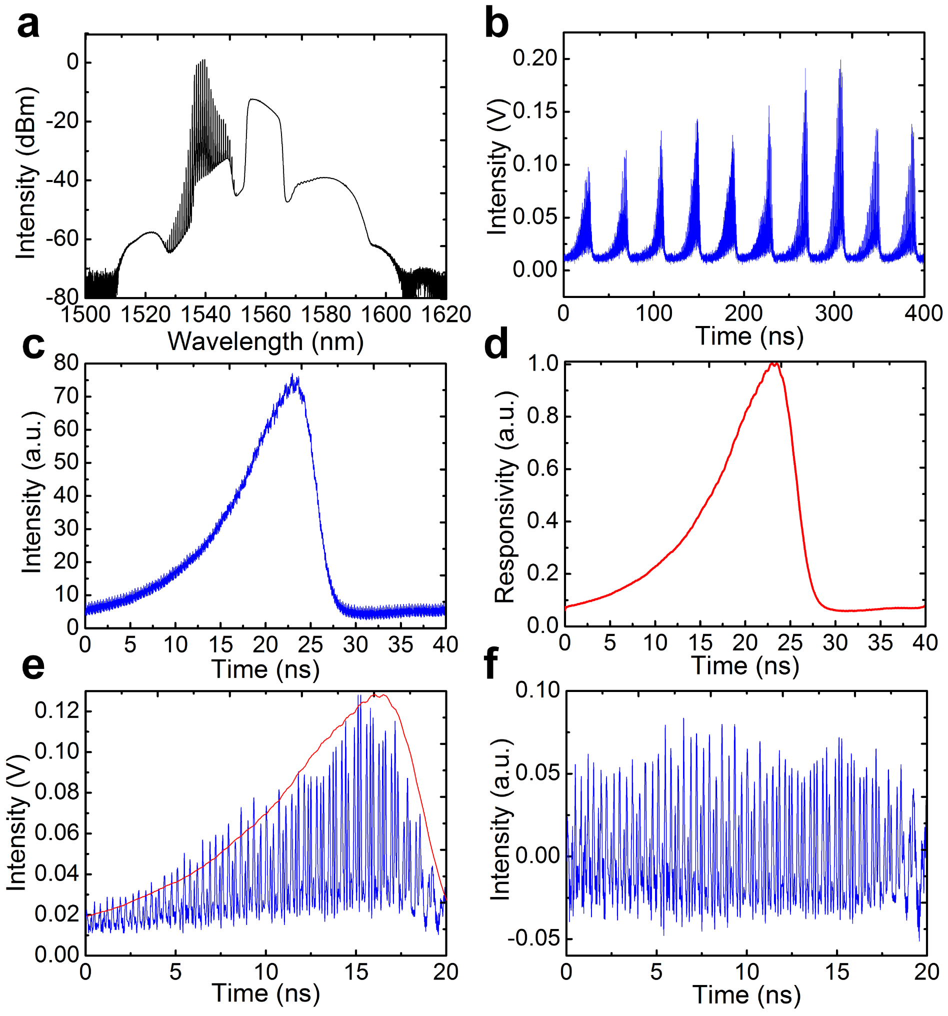

Supplementary Figure 3 | Measurement and calibration of dynamic waveform. a, four-wave mixing (FWM) spectrum for the dynamic waveform measurement after the highly-nonlinear fiber (HNLF). **b,** Output waveform from the panoramic-reconstruction temporal imaging (PARTI) system. For each buffered signal-under-test (SUT), system will output 10 frames of waveform, which correspond to 10 consecutive positions on the buffered SUT. **c,** The waveform after adding up 2500 output frames. **d,** Smoothed and normalized curve from **c**, which represents the responsivity across the input time-window and is used to calibrate the intensity of output waveforms. **e,** The zoom-in of the fifth frame in **b** (blue curve) together with the calibration curve (red curve). **f,** The waveform after intensity calibration.

Waveform stitching

Supplementary Figure 4 describes the process for waveform stitching, which consists of three main steps: initial temporal alignment, stitching-area identification and fine temporal alignment. In step 1, two consecutive frames of magnified waveforms (blue and red curves) that correspond to the evolution time 0.73 ns~1 ns in main text Figure 5e (case 2) are shown in Supplementary Figure 4a as an example. Each section contains 2000 data sampling points, corresponding to 20 ns on the magnified waveform and 320 ps on the original SUT. The initial alignment is made according to the predefined scanning step size in stroboscopic acquisition, i.e. 150 ps in this work. Considering the temporal magnification ratio of 61.5, a step size of 150 ps on the SUT corresponds to about 9.23 ns, or 923 data points on the magnified output waveform. Therefore, the blue trace and red trace are relatively shifted accordingly as initial temporal alignment. Vertical offset is used for easier waveform comparison. As observed in the zoom-in view of the temporally overlapping area (1077 data points) of the two traces in Supplementary Figure 4b, the repetitive three-pulse structure

in two traces already matches well with each other, which in turn confirms the accuracy of the initial alignment. Ideally, the overlapping areas in both traces should appear identical, such that any point within the area can serve as the stitching point between the two traces. However, because of the non-uniform responsivity of the time lens, the trailing area of the blue trace as well as the leading area of the red trace show larger distortion on the waveform owing to poorer signal-to-noise ratio (SNR). Consequently, an optimal stitching area exists where both traces have reasonable SNR and thus exhibit highest resemblance. Therefore, in step 2, the optimal stitching area is identified by calculating the cross-correlation coefficient between the two traces. Note that the cavity roundtrip time of the microresonator is about 11.3 ps, which corresponds to about 70 data points on the magnified waveform. Therefore, the overlapping areas in both traces can be segmented into 15 sections according to the roundtrip time of the microresonator, which function as 15 comparison pairs. The maximum values of the cross-correlation function between each pair of waveform sections are calculated to identify the optimal stitching area as shown in Supplementary Figure 4c. During the calculation, a comparison window is slightly expanded to 80 data points such that slight temporal misalignment between the two traces will not affect the maximum cross-correlation coefficient. As observed in Supplementary Figure 4c, the coefficient first rises and then falls, which matches well with the SNR evolution. The maximum value is located at 10th roundtrip and it is therefore selected as the stitching area, denoted by the green dashed region in Supplementary Figure 4b. In the final step, fine temporal alignment is achieved according to the cross-correlation function to locate the precise stitching point. The cross-correlation function between the 10th waveform sections in both traces is shown in Supplementary Figure 4d. The maximum correlation value is reached when the two waveforms are relatively shifted for one data point (labelled by green triangle), which indicates that the red trace should be

shifted by one data point for fine temporal alignment during the data stitching. To avoid splitting the single roundtrip waveform during the stitching, the starting point of the 10th roundtrip waveform is used as the stitching point, and the data from the two traces are combined accordingly, which generates the stitched waveform shown in Supplementary Figure 4e. Since the SNR evolution is similar for the overlapping areas between any two neighbouring frames, the approach above is adapted throughout the rest of the data processing. Just like the process demonstrated above, for each buffered SUT, the ten magnified waveforms can be stitched together to reconstruct a 1.5-ns long waveform (de-magnified time scale), which depicts the dynamic dissipative-soliton-evolution process with a temporal resolution of 740 fs, representing a time-bandwidth product about 5 times larger than the previous record value demonstrated in conventional temporal imaging systems. To visualize the roundtrip waveform evolution process, the 1.5-ns long waveform is sectioned according to the roundtrip time, i.e. 11.3 ps, and the 2D evolution map shown in Figure 5 in the main text can be obtained.

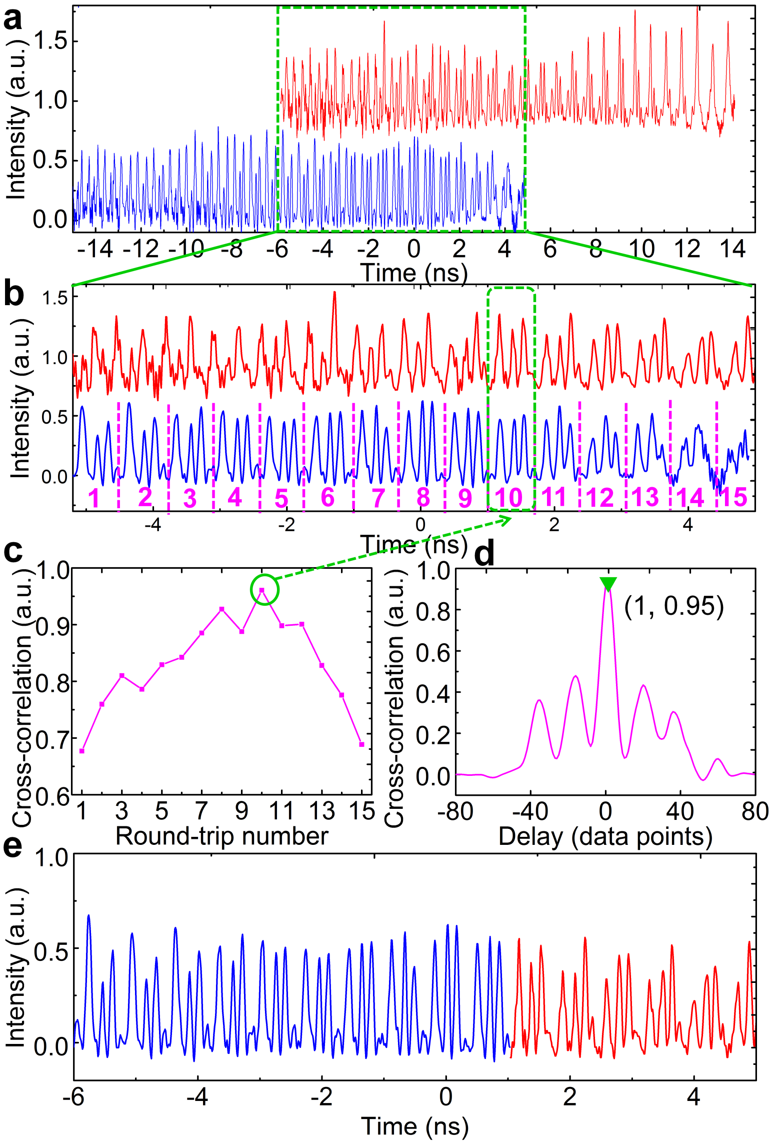

Supplementary Figure 4 | Waveform stitching process. a, The magnified waveform in two consecutive output frames (blue, red) are temporally aligned according to the scanning step size in stroboscopic acquisition and are vertically offset for comparison. **b,** Zoom-in of the temporal overlapping area in **a**. Each trace is segmented into 15 sections according to the roundtrip time of the microresonator, which serve as 15 comparison pairs. **c,** The maximum values of cross-correlation coefficients between each comparison pair in the blue and red traces. The maximum value labelled by the green circle indicates the optimal stitching area, while the lower values are

attributed to the low SNR in either red or blue trace. **d,** The cross-correlation function between the 10th waveform sections in blue and red traces. The horizontal location of the peak indicates the amount of shift required for fine temporal alignment. **e,** Longer waveform after stitching according to the identified stitching point.

Supplementary Note 4: Estimation of scalability.

Owing to the requirement for repetitive amplification to overcome the loss inside the optical buffer, the amplified spontaneous emission (ASE) noise will accumulate, which degrades the optical SNR of the signal. As this effect is universal for all amplifiers, the SNR degradation is unavoidable, which will limit the ultimate scalability of the PARTI system. To find out the maximum allowable buffering number of times and thus the ultimate performance of the PARTI system, the noise accumulation inside the buffer is theoretically analysed.

The repetitive amplification inside an optical buffer is essentially equivalent to a long-haul transmission system, where erbium-doped fibre amplifiers (EDFAs) are deployed periodically to compensate the transmission loss. In fact, circulating loop transmission experiments have been conducted to study the long-haul transmission system [2]. Typically in the long-haul transmission system, the EDFA gain is just enough to compensate the fibre loss in each section such that the signal intensity remains relatively constant, which matches exactly with our case inside the optical buffer. Therefore, the total ASE power P_{sp} after N times of buffering can be expressed by [3]:

$$P_{sp} = 2n_{sp}h\nu_0 N\left(G-1\right)\Delta\nu_{opt} \quad (3)$$

where h is the Planck's constant, ν_0 is the central frequency of the buffered signal, N is the buffering time, G is the gain of the intra-buffer amplifier and $\Delta\nu_{\mathrm{opt}}$ is the bandwidth of the optical filter after the amplification. n_{sp} is the population inversion factor and can be expressed as $n_{\mathrm{sp}} = \frac{N_2}{N_2 - N_1}$, where the N_2 and N_1 are atomic populations for the excited and ground states of erbium, respectively. The precise value of n_{sp} can be found by solving the rate equations [3], which varies along the EDF and depends on the pump and signal power. While it is beyond our scope to find the exact value, it can be conveniently estimated by

$$n_{\mathrm{sp}} \approx \frac{1}{2} F_{\mathrm{n}} \tag{4}$$

where F_{n} is the noise figure of EDFAs [3]. The state-of-the-art pre-amplifiers can achieve a noise figure of 4.3 dB (e.g. Amonics AEDFA-PA-30). Therefore, the value of n_{sp} is adopted accordingly to be 1.346. In addition, according to the current buffer parameters, G is 15.85 (12dB) and $\Delta\nu_{\mathrm{opt}}$ is 1.25 THz (10 nm). $h\nu_0$ is around 0.8 eV at 1.55 μm. Inserting these values into equation (3), it is obtained that the accumulated noise power after 100 times of buffering (N=100) is about 0.64 mW. Since this power level is unlikely to saturate the EDFA, the gain can be regarded as constant for the signal so as to keep the same signal power in each circulation. According to our typical experimental value, the average intra-cavity signal power is 1.25 mW, which is comparable to the accumulated ASE power. However, note that the signal has a temporal duty ratio of around 1/8 (5ns versus 40-ns cavity time), while the ASE power is evenly distributed over time. Consequently, the peak power of signal power is 10 mW and therefore the optical SNR after 100-time buffering is about 12 dB based on the theoretical analysis shown above. Meanwhile, the optical SNR is not

the only parameter determining the final signal quality obtained from the oscilloscope. According to [3], the electrical SNR is related to the optical SNR as

$$SNR_{\mathrm{el}} \approx \frac{\Delta \nu_{\mathrm{opt}}}{2\Delta f} SNR_{\mathrm{opt}} \tag{5}$$

if only the dominating signal-spontaneous beat noise is considered, where Δf is the detection bandwidth of the photodetector. To simplify the analysis, we regard the temporal magnification system and the photodetector as a single unit, which achieves a detection bandwidth of around 500 GHz. The small optical SNR degradation inside the temporal imaging system can be neglected compared to that induced by the optical buffering, since only one stage of parametric conversion and a single low-noise pre-amplifier are involved. Under this circumstance, the electrical SNR of the final signal is estimated to be around 13 dB, slightly lower than the standard criteria (15.6 dB) in optical communication, which guarantees a bit-error rate (BER) of 10^{-9} [3]. According to this criterion, the maximum allowable buffering number of times is calculated to be 53. On the other hand, it is also worth noting that the PARTI system operates with high-speed real-time oscilloscopes. For a 20-GHz real-time oscilloscope (same as the one used in the experiment), the effective number of bits (ENOB) is typically about 6 [4], which set the effective dynamic range of signal acquisition at 15 dB. Therefore, specifically for the PARTI system, an electrical SNR of 15 dB is sufficient. Under these circumstances, the maximum allowable buffering number of times is 61.

To realize the 100 times of buffering, the buffer requires further improvement in terms of cavity loss. Currently the intra-cavity variable optical delay line is constructed with a circulator, a fibre collimator, and a mirror mounted on a translation stage, which together induce an attenuation of about 3.5 dB. This is required for finding the optimal operating condition, e. g. the temporal

scanning step size. However, when the optimal cavity length is fixed, the variable delay line can be readily replaced by an SMF. In addition, the intra-cavity amplitude modulator (AM) can be replaced by state-of-the–art high-speed optical switches (http://eospace.com/switches.htm) that offers insertion loss of less than 3 dB. Therefore, it is expected that the cavity loss of the optical buffer can be further reduced to around 6 dB, which further reduces the ASE noise by lowering the required gain. Under this circumstance, the SNR is calculated to be around 20 dB after 100 times of buffering, which is sufficient to generate high-fidelity results.

Our simplified estimation matches with the comprehensive derivation in [5], where it is shown that the SNR can be slightly above 15.6 dB after 100 circulations for an input power of -10 dBm at 160 $Gbit \cdot s^{-1}$ data rate. Moreover, in a pulse replication experiment [6], an optical buffer with a similar configuration has successfully generated more than 1,000 replicas of single pulse with SNR > 20dB, which further convince us the potential scalability of PARTI system to be at least 100 times.

Supplementary References:

1. Diels, J. & Rudolf W. Ultrashort Laser Pulse Phenomena, Second Edition (Massachusetts, Academic Press, 2006).
2. Bergano, N. S. & Davidson, C. R. Circulating loop transmission experiments for the study of long-haul transmission systems using erbium-doped fiber amplifiers. *J. lightwave Technol.* **13**, 879-888. (1995).
3. Agrawal, G. P. Fiber-Optic Communication Systems, Third Edition (A John Wiley & Sons. Inc., Publication, 2002).

4. Khilo, A. *et al.* Photonic ADC: overcoming the bottleneck of electronic jitter. *Opt. Express* **20**, 4454-4469. (2012).

5. Langenhorst, R. *et al.*. Fiber loop optical buffer. *J. Lightwave Technol.* **14**, 324-335 (1996).

6. Jolly, A., Gleyze, J. F. & Jolly, J. C. Static & synchronized switching noise management of replicated optical pulse trains. *Opt. Commun.* **264**, 89-96 (2006).
